# Airbnb and neighborhood crime: The incursion of tourists or the erosion of local social dynamics?

**DOI:** 10.1371/journal.pone.0253315

**Published:** 2021-07-14

**Authors:** Laiyang Ke, Daniel T. O’Brien, Babak Heydari

**Affiliations:** 1 School of Public Policy and Urban Affairs, Northeastern University, Boston, MA, United States of America; 2 Department of Mechanical and Industrial Engineering, Northeastern University, Boston, MA, United States of America; 3 Boston Area Research Initiative (BARI), Northeastern University, Boston, MA, United States of America; 4 Network Science Institute, Northeastern University, Boston, MA, United States of America; Xiamen University, CHINA

## Abstract

The proliferation of internet-based home-sharing platforms like Airbnb has raised heated debates, with many in the general public believing that the presence of Airbnb listings can lead to an increase in crime and disorder in residential neighborhoods. Despite the importance of this debate to residents, policymakers, and other stakeholders, few studies have examined the causal linkage between Airbnb listings and crime in neighborhoods. We conduct the first such empirical test in Boston neighborhoods, focusing on two potential mechanisms: (1) the inflow of tourists might generate or attract crime; and (2) the creation of transient properties undermines local social dynamics. Corresponding to these mechanisms, we examine whether the number of tourists (approximated with reviews) or the prevalence of listings predict more incidents of private conflict, social disorder, and violence both concurrently and in the following year. We find evidence that increases in Airbnb listings–but not reviews–led to more violence in neighborhoods in later years. This result supports the notion that the prevalence of Airbnb listings erodes the natural ability of a neighborhood to prevent crime, but does not support the interpretation that elevated numbers of tourists bring crime with them.

## Introduction

The expansion of internet-based short-term rental platforms like Airbnb has raised heated debates in recent years. Airbnb enables travelers and visitors to stay in idle private residential properties as an alternative to hotels. Consequently, it creates an inflow of tourists into residential neighborhoods without hotels where they were previously unlikely to go, potentially causing undesirable impacts (aka negative externalities) for these neighborhoods [1]. One of the concerns held by some in the general public and presented in multiple media reports is that the presence of Airbnb listings can lead to an increase in crime and disorder in a neighborhood. For example, an article in 2016 in the New York Times reported that residents in New Orleans were distraught at Airbnb guests’ disruptive behaviors [2]. The story resulted in a city-wide request for stricter regulations on home-sharing activities. Another article from Splinter News told a broader story of how sharing economy platforms like Uber and Airbnb are exploited by criminals [3]. Similar concerns have even given rise to websites like AirbnbHell.com, which documents the dangers of using Airbnb services. However, despite a number of media claims and anecdotal evidence, few studies have examined the causal linkage between Airbnb listings (or short-term rentals more generally) and crime in neighborhoods, and those that have have done so largely descriptively [4]. Thus, there remains a need for a robust empirical test of this relationship that can inform residents, policy makers, and other stakeholders.

### Short-term rentals and crime: Two potential mechanisms

Most of the discussions about short-term rentals and crime in neighborhoods rest on the logic that tourists might bring such issues, a relationship that has been investigated more generally by researchers in both criminology and tourism. Often, this relationship is framed in terms of routine activities theory [5], in which a crime is understood as requiring three minimal elements: a motivated offender, a suitable target, and the lack of a guardian. There are three hypotheses that arise from this framing. Ryan (1993) makes the case for two of these. One is that tourists make for suitable targets, either because they are known to have money on them or are more vulnerable when navigating an unfamiliar city. Second, he argues that because tourist locations are known to have many suitable targets, they attract more potential offenders, putting both tourists and residents at greater risk [6]. There is more evidence for the first of these two hypotheses, as at least three studies have found that tourists are more likely to be victimized than locals [7–13]. Third, some have noted that tourists might engage in criminal or disruptive behavior themselves. For example, Boivin and Felson (2018) found that urban neighborhoods with more visitors feature elevated rates of crime committed by visitors but no increase in crimes committed by locals [14]. Similarly, arguments against short-term rentals often hinge on the assumption that tourists might bring drunkenness or other unruly behavior with them. Such behaviors are more frequent in downtown areas and business districts with many shops, restaurants, and bars, but would be less familiar in a residential neighborhood that now has many short-term rentals [15].

We also note a second mechanism by which short-term rentals might impact neighborhood crime, one that is less prevalent in public discussions. It draws off of the sociological\criminological concept of social organization–that is, neighborhoods whose residents know and trust each other and share common values are more able to establish and enforce social norms [16]. In turn, they tend to have lower levels of crime [17]. One of the main factors that inhibits a strong social organization is residential instability, because it is hard to develop relationships and establish norms if a sizable proportion of the population is transient [18]. It would stand to reason, then, that if a sufficient number of units throughout a community have been converted to short-term rentals–the most transient form of occupancy possible–it can undermine the social organization and its ability to discourage and prevent crime. A strong social organization is also associated with and able to support various dynamics and processes subsumed under the term ‘social capital,’ including trust, reciprocity, and social cooperation [19]. Further, researchers focusing more on this latter set of terminologies has repeatedly found that numerous manifestations of social capital are associated with lower incidence of crime [20, 21]. Moreover, previous theoretical work have demonstrated an strong impact of community structure (measured by network modularity) on population level attributes such as cooperation, fairness and stability [22–26].

We then have two potential mechanisms by which short-term rentals can lead to increased crime in a neighborhood–by bringing tourists who then perpetrate crime and disorder, or by creating transience that undermines local social dynamics that might in turn mitigate or prevent crime. It is important to note that these mechanisms are not mutually exclusive and could be operating simultaneously. That said, we note two analytic considerations that might disentangle their presence. The first consideration is temporal. If issues generated by the prevalence of short-term rentals arise from the presence of tourists themselves, we would anticipate increases in Airbnb listings and crime to be nearly if not perfectly concurrent. In contrast, if an abundance of listings is undermining the social organization of the community and its natural ability to prevent and discourage crime, then there would be a more gradual erosion. In this case we would expect to see any effect of Airbnb listings on crime be lagged, increasing over time. The second consideration regards the way we measure the presence of Airbnb in a community. If tourists themselves are perpetrating crime and disorder, the focus should be on the quantity of tourists listings are bringing to the neighborhood, rather than the listings themselves. Alternatively, if the concern is transience, we will want to focus on the quantity of listings. We describe our measurement strategy for each in the next subsection.

### Previous evidence and the current study

Whether those staying in Airbnb listings attract or perpetrate crime, or, alternatively, a large number of Airbnb listings undermine the social organization of the community, it has become a common perception that the rise of short-term rentals in a residential neighborhood will be accompanied by a rise in crime. This notion has only been examined by two empirical studies, though neither directly tests this causal claim. One study looking at the association only examined the correlation between crime and Airbnb listings and did not control for other neighborhood characteristics nor the temporal relationship between the arrival of Airbnb listings and shifts in the crime rate [4]. Another paper used policy implementations as a natural experiment, but analyzed only at the citywide scale [27].

Here we fill this gap in the literature by testing whether the presence of Airbnb leads to increases in crime across the neighborhoods of Boston, MA. As noted above, we use two measurement strategies to study the link between short-term rentals and crime. First, we quantify the influx of Airbnb-related tourists by tabulating reviews for Airbnb listings in the neighborhood. The measure of *usage* is drawn from [29]. Our second strategy focuses on the listings in a neighborhood, for which we employ two such measures. The more common measure in the literature is what we refer to as *density*, which is the number of listings divided by the total number of households. This measure is one step forward to what we expect to impact neighborhood social organization. However, it does not take into account the geographic distribution of these listings. To illustrate, consider two neighborhoods with the same number of households and the same number of Airbnb listings. In one, the listings are distributed throughout the neighborhood, in the other, they are concentrated in two condo buildings that have been effectively converted into unofficial hotels. It would seem likely that the former would have a more pernicious impact on the neighborhood’s social networks by undermining relationships more broadly, whereas the impacts of the latter would be more contained at a handful of properties. Thus, we also create measure we refer to as *penetration*, which is defined as the proportion of buildings in the neighborhood with Airbnb listings. This better captures how Airbnb listings are distributed through the community, potentially better capturing how likely they are to impact the social organization. As described above, an association between usage and crime would be evidence that tourists are generating or attracting crime and disorder themselves. Meanwhile, if penetration or density are predictive of crime and disorder and usage is not, there is a stronger case that an abundance of listings in a neighborhood are undermining the social organization.

We examine the relationships between the measures of Airbnb usage, penetration, and density and three types of social disorder and crime: public social disorder (e.g., drunkenness, loitering), private conflict (e.g., landlord-tenant disputes, vandalism), and violence (e.g., fights), all per 1,000 persons in a neighborhood. This allows us to examine in a nuanced way the nature of the impact that short-term rentals might have on neighborhoods. We use fixed effects models to conduct these analyses, comparing the relationships between these variables from 2011–2017, as Airbnb went from a minor to more major factor in Boston neighborhoods. As noted above, the two mechanisms by which short-term rentals might impact neighborhoods–either the tourists generating or attracting crime themselves, or the prevalence of listings eroding the social organization–would operate on different time scales. If the presence of tourists is responsible for crime, we would anticipate the impacts to occur in the same year as the increase of usage. The erosion of the social organization would take more time to result in elevated crime, lagging increases in listings by one or more years. Thus, we run the difference-in-difference fixed effects models with the Airbnb measures as measured concurrently with the crime outcome measures, with a one-year lag between the Airbnb measures and crime and disorder, and then with a two-year lag. Importantly, this work adds a rigorous empirical perspective to the ongoing debate regarding the negative externalities of short-term rental platforms such as Airbnb.

## Data and methods

### Measuring Airbnb presence

We use the period between 2011 to 2018 to quantify the presence of Airbnb in Boston. To estimate the presence of Airbnb in a neighborhood, we obtained datasets from InsideAirbnb.com, an independent, non-commercial website that scrapes and publishes longitudinal Airbnb listings’ records for cities across the world for the purpose of research. InsideAribnb.com has published these data annually since 2015, but Airbnb entered Boston in 2009. In order to overcome this limitation, we leveraged the “host since” field, which indicates the date a property became an Airbnb listing, to estimate which Airbnb listings were present in each year 2011–2014. Koster et al. (2018) took a similar approach using the date of a listing’s first review, but we found that the “host since” variable more consistently had a value and would be more precise in any case. InsideAirbnb.com also publishes a separate dataset on the reviews received by each listing along with the listings data [28]. The reviews datasets have been used to estimate the amount of tourists brought by Airbnb services [29, 30]. We note that although we consider the start year of our study as 2011, there were still some Airbnb units in Boston as early as 2008 that are not considered in this study. This should not impact the results given the limited nature of this presence; however it might have implications for testing pre-treatment parallel trends in the DID analysis as we will explain in the *Robustness Check* Section.

Following the practice of Horn & Merante (2017), we use census tracts to approximate neighborhoods (avg. population = 4,000; 168 with meaningful population in Boston). We then linked listings to the containing census tract, allowing us to calculate neighborhood-level measures of Airbnb’s prevalence. Though listings are not necessarily geographically precise, InsideAirbnb.com indicates that listings are 0–450 feet from the actual address. Meanwhile, census tracts cover .5 mile radius, meaning that most listings should fall in the appropriate census tract.

We use three measures to quantify the level of Airbnb presence in each tract. Specifically, these aim to operationalize the quantity of listings and the quantity of tourists they bring to the neighborhood. For listings, our primary measure *penetration* sought to capture how they were spatially distributed across the neighborhoods. It was calculated as the number of unique addresses with listings divided by the number of parcels (lots that contain one or more units, per the City of Boston’s Assessing Department) in the census tract, thereby approximating the number of buildings with at least one Airbnb listing. This might be a more appropriate proxy, for instance, when Airbnb listings are many in a neighborhood but concentrated in one or two condo buildings, thus geographically constraining their overall impact. For robustness, we also measured *density*, or the ratio of Airbnb listings to housing units. This measurement has been widely adopted in previous studies on Airbnb [31, 32]. The quantity of tourists attracted was operationalized as *usage*, calculated as the number of reviews divided by housing units in a census tract as recommended by Schild (2019) [29].

### Using 911 call data to measure crime activity

We utilized three variables measuring crime and disorder developed by the Boston Area Research Initiative from 911 dispatches from 2011–2018. These measures were calculated as the rate per 1,000 residents of events falling into a pre-determined set of categories from the dispatches. They include: public social disorder, including intoxicated individuals, lewdness, and drunken disturbances; private conflict includes issues like landlord/tenant trouble, breaking and entering, and vandalism; and violence includes events like armed robberies, assaults, a person with knife, and fights.

### Estimation strategies

The key research question we ask in this study is whether the proliferation of Airbnb in a neighborhood lead to higher level of crime events in that neighborhood. The panel dataset we assembled at the census tract-level allows us to employ a generalized multiple time period, multiple group Difference-in-Difference (DID) design, in which Airbnb presence acts as a continuous “treatment”, predicting changes in crime in a neighborhood.

The estimated equation is:

$$\begin{array}{c} Y_{i,t}=\alpha +\gamma Airbnb_{i,t-\tau }+\delta X_{i,t}+\eta_{i}+\beta_{t}+\varepsilon_{i,t} \end{array}$$ (1)

where *i* represents the census tract, *t* represents the year, and *τ* is used to introduce time lag and lead for the treatment variable. *Y*_*i*,*t*_ is the crime level measured by the number of private conflict, social disorder, and violence events per 1,000 people, *X*_*i*,*t*_ is a vector of time-variant neighborhood-level controls, and *γ* is the estimated causal effect of Airbnb presence. *η* and *β* are the neighborhood (tract) and year fixed effects, respectively, capturing both time-invariant characteristics of tracts and spatially-invariant characteristics of years (for example, a city-wide increase in Airbnb prevalence or crime level). We report the results based on using *income* as the main tract-level control variable, although we test a number of other controls for robustness test. *Income*_*i*,*t*_ measures the median household income (drawn from the American Community Survey’s five year estimations at the census tract-level, appropriate to the year in question. We estimate Eq (1) using deviation from mean approach, and standard errors are clustered at the tract level.

To further test the direction of causality for the results, we use a lag/lead analysis in the spirit of Granger [33, 34]. This method is used when the sample includes multiple years and uses both lead and lagged versions of the treatment variable (*τ* can be both positive and negative).

## Results

### Descriptive analyses

**Fig 1:**
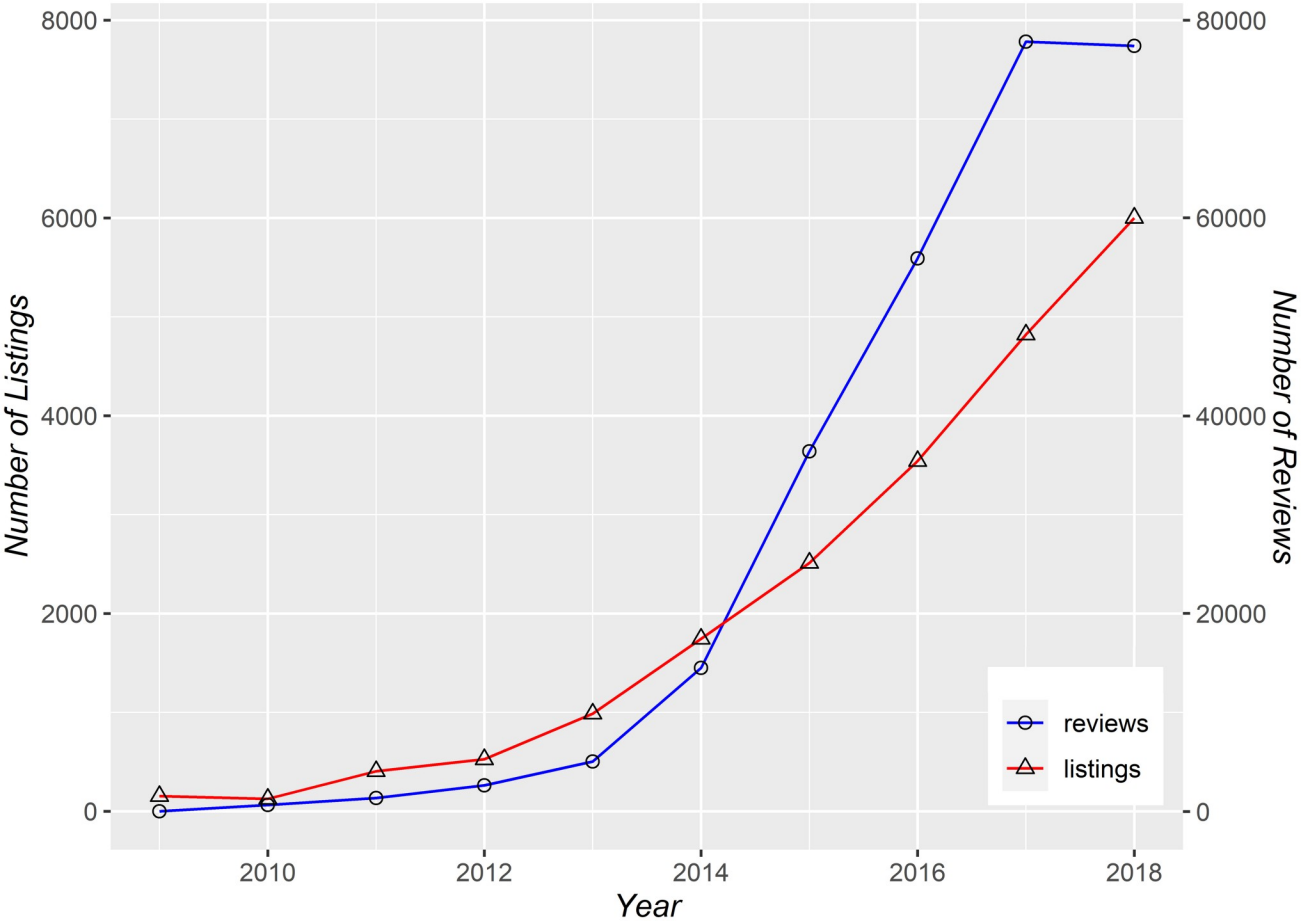
Airbnb’s expansion in Boston. The number of Airbnb listings and reviews in Boston between 2009 and 2018.

**Fig 2:**
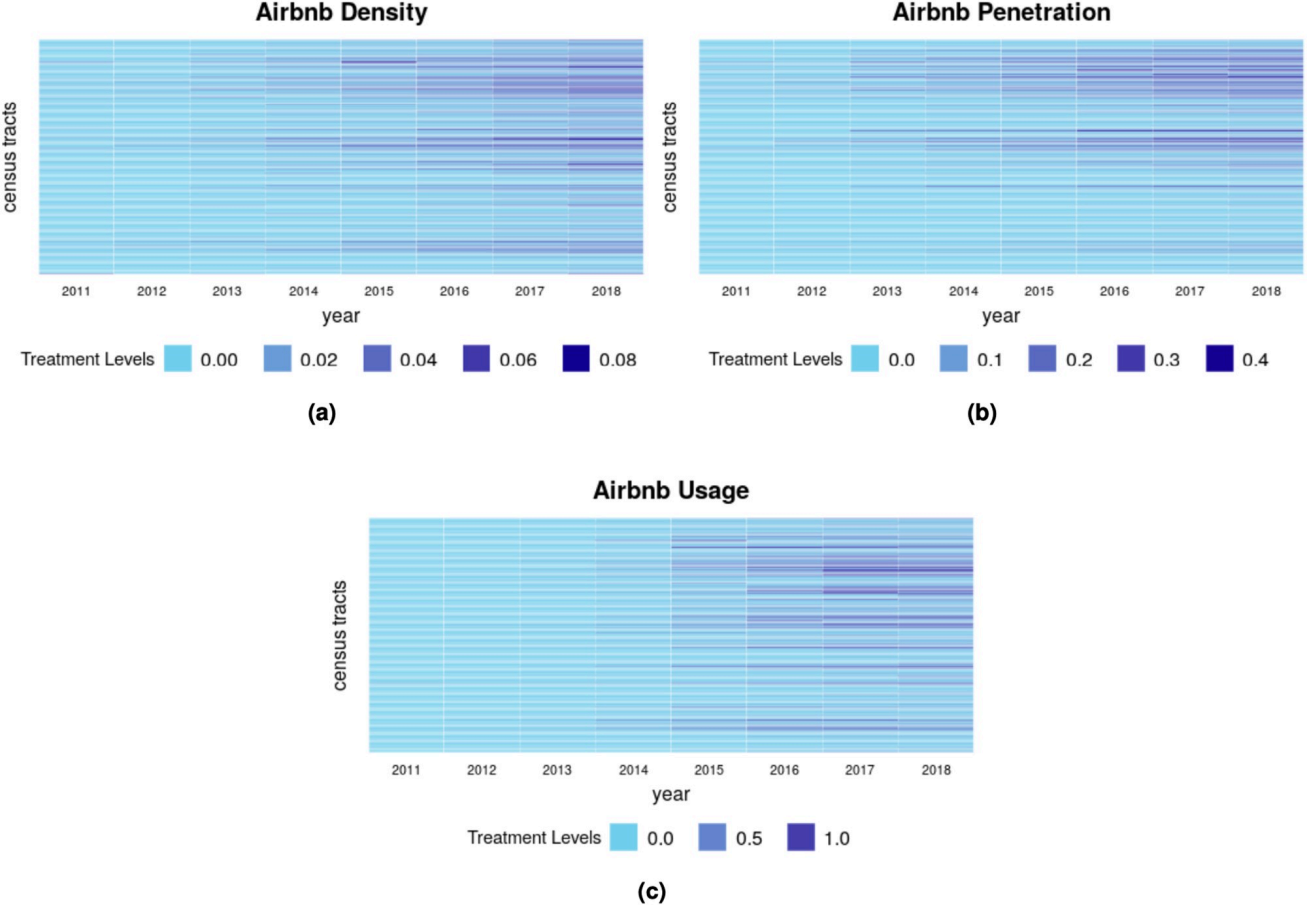
Airbnb’s presence in Boston. (a) Airbnb density, (b) Airbnb penetration, and (c) Airbnb usage. Each row represents a census tract from 2011 to 2018. The darker the color, the higher the Airbnb presence. Tracts are in the same position in each panel, meaning we can compare panels to confirm that most tracts with high level of presence on one measure scored similarly on the other measures.

Before testing our main question, it is useful to examine the growth and distribution of Airbnb activities in Boston. As depicted in Fig 1, Airbnb had limited presence in Boston at first, with a negligible number of listings and reviews before 2014. There was rapid growth, however, between 2014 and 2018, over which time the number of listings more than doubled from 2,558 to 6,014. There were also nearly 80,000 total reviews by 2018. That is not to say, however, that this growth was uniform across neighborhoods. Certain census tracts were the first to have a measurable presence of Airbnb and then proceeded to have high levels of Airbnb listings. Fig 2 shows how Airbnb services increased from 2010 to 2018 and across census tracts in Boston. We focus on two main measures to capture Airbnb activities: penetration, or the proportion of buildings with at least one listing; and usage, or the number of reviews per housing unit in the neighborhood. As indicated in Fig 2a, by 2018, the tracts with the highest penetration of Airbnb had listings in as many as 40% of buildings. Likewise, the neighborhoods with the highest level of usage had as many as one review per housing unit. In contrast, in many other tracts the presence of Airbnb was limited or even absent throughout the study period. Meanwhile a handful of tracts started with very low Airbnb presence and then witnessed rapid growth of Airbnb-related activities.

**Fig 3:**
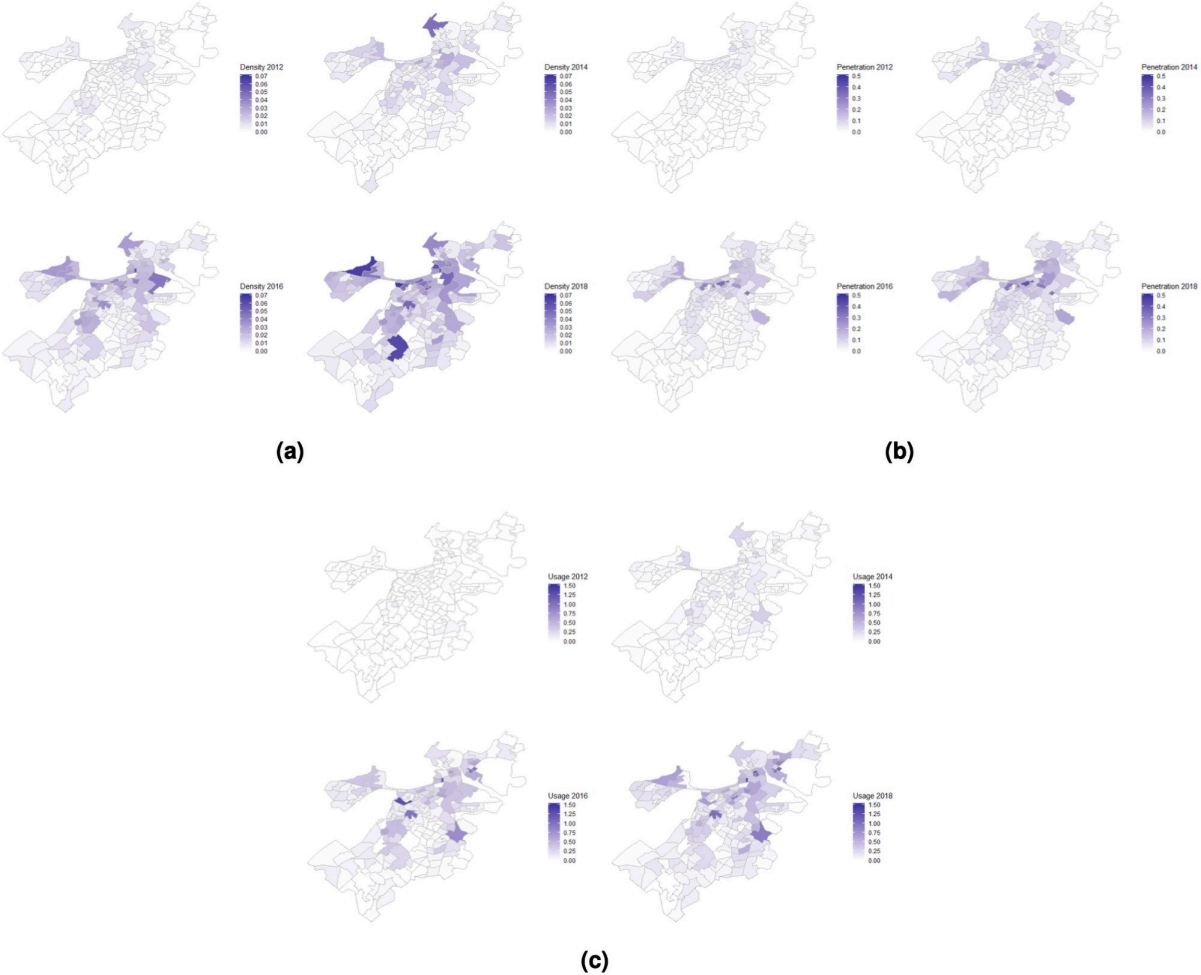
Evolution of spatial distributions of Airbnb in Boston. (a) Airbnb density, (b) Airbnb penetration, and (c) Airbnb usage in 2012, 2014, 2016, and 2018.

Fig 3 maps the spatial distributions of the three measures of Airbnb supply over time. For Airbnb density (Fig 3a), we see that census tracts in the urban center (northeast on the map) show relatively high Airbnb presence from the beginning, but that in recent years the tracts with the highest level of Airbnb penetration emanate further out into surrounding, more residential neighborhoods.

### The concurrent and lagged impacts of Airbnb on crime

We use difference-in-difference models (Eq (1)) to test whether a rise in the prevalence of Airbnb in a census tract in one year predicts increases in crime and disorder in the following year. We focus on two ways in which short-term rentals can impact a neighborhood. The first is through two measures of the quantity of listings in a neighborhood: the penetration of Airbnb, measured as the proportion of buildings with at least one listing; and the density of Airbnb, or the ratio of listings to total households. We believe the latter is the stronger measure for our purposes (see Introduction for more), but include both as a check. The second strategy is to capture the amount of tourists brought in by listings via the measurement of usage, or the ratio of user reviews to households. The model outcomes include three measures of crime and disorder: private conflict between people who live together, like landlord-tenant disputes; public social disorder, like drunkenness and noise complaints; and public violence, including fights (see Methods). The models control for tract-level and year fixed effects. In order to make the parameter estimates that follow more interpretable, we note that the average census tract in the average year experienced 11.32 events of private conflict, 7.68 events of public social disorder, and 28.58 events of public violence per 1,000 residents.

We begin by testing the relationship between Airbnb prevalence and crime in the same year (See Table 1). We see only one significant effect, which is Airbnb penetration predicting higher levels of violent crime (*β* = 0.328, *p* < 0.05). Otherwise, density and usage were not associated with any forms of crime, nor were social disorder or private conflict associated with any of the Airbnb measures.

**Table 1:** Same-year DID regressions on social disorder and crime.

	Events of Private Conflict	Events of Social Disorder	Events of Violence
Airbnb Density (%)	-0.207	0.080	1.226
	(0.207)	(0.285)	(0.621)
Airbnb Penetration (%)	0.005	-0.004	0.328*
	(0.035)	(0.073)	(0.133)
Airbnb Usage (%)	0.000	-0.004	0.025
	(0.008)	(0.011)	(0.021)
Tract FE	Yes	Yes	Yes
Year FE	Yes	Yes	Yes
Controls	Yes	Yes	Yes
Observations	1171	1171	1171
F (Density)	0.88	1.20	2.17
F (Penetration)	0.36	0.97	3.13
F (Usage)	0.36	0.93	0.77

Note: clustered standard errors are displayed in parenthesis. Control variable is median household income. The average census tract in the average year experienced 11.32 events of private conflict, 7.68 events of public social disorder, and 28.58 events of public violence per 1,000 residents. Significance levels:

* p<0.05;

** p<0.01;

*** p<0.001.

We then compare these results to models that test the relationship between Airbnb measures from the previous year on crime (i.e., one-year lags). In these models, neighborhoods with a higher level of Airbnb penetration saw rises in violent crime in the following year (*β* = 0.546, *p* < 0.0001), and notably to a greater extent than the concurrent measure of penetration. There was still no corresponding effect on public social disorder or private conflict, however. Airbnb density in the previous year was also associated with higher levels of violent crime, albeit at a lower significance, and thus magnitude, relative to penetration ((*β* = 1.407, *p* < 0.05). Airbnb usage had no effect on any of the three measures in the following year(Table 2).

**Table 2:** One-year lagged independent variables.

	Events of Private Conflict			Events of Social Disorder			Events of Violence		
	(1)	(2)	(3)	(4)	(5)	(6)	(7)	(8)	(9)
Airbnb Penetration (lag 1)	0.041			-0.115			0.546***		
	(0.039)			(0.118)			(0.133)		
Airbnb Density (lag 1)		-0.112			-0.426			1.407*	
		(0.227)			(0.293)			(0.614)	
Airbnb Usage (lag 1)			0.001			-0.011			0.037
			(0.009)			(0.016)			(0.021)
Tract FE	Yes	Yes	Yes	Yes	Yes	Yes	Yes	Yes	Yes
Year FE	Yes	Yes	Yes	Yes	Yes	Yes	Yes	Yes	Yes
Controls	Yes	Yes	Yes	Yes	Yes	Yes	Yes	Yes	Yes
Observations	1004	1004	1004	1004	1004	1004	1004	1004	1004
F	0.62	0.16	0.04	0.8	1.32	0.79	8.7	2.69	1.56

Note: clustered standard errors are displayed in parenthesis. Control variable is median household income. The average census tract in the average year experienced 11.32 events of private conflict, 7.68 events of public social disorder, and 28.58 events of public violence per 1,000 residents.

Significance levels:

* p<0.05;

** p<0.01;

*** p<0.001.

If the increase in crime rate is driven by changes in social organization, we expect to see the effect to persists and possibly strengthen over a more extended period of time. To further test the validity of this mechanism,we repeated the previous analysis, this time with a two-year lag on independent variables.

Results of the two-year lagged analysis are in general agreement with those with one-year lag in terms of the impact of Airbnb penetration on events of violence. Moreover, Airbnb penetration not only predicted increased violence at this time scale, but also showed a moderate impact on events of private conflict (*β* = 0.097, *p* < 0.05), an effect that was not present in the one-year lagged analysis. The effects of Airbnb usage and density also concurred with the one-year lagged analysis (Table 3).

**Table 3:** Two-year lagged independent variables.

	Events of Private Conflict			Events of Social Disorder			Events of Violence		
	(1)	(2)	(3)	(4)	(5)	(6)	(7)	(8)	(9)
Airbnb Penetration (lag 2)	0.097*			-0.162			0.553***		
	(0.041)			(0.107)			(0.119)		
Airbnb Density (lag 2)		0.039			-0.884			1.167*	
		(0.215)			(0.472)			(0.529))	
Airbnb Usage (lag 2)			0.014			-0.036			0.037
			(0.013)			(0.029)			(0.027)
Tract FE	Yes	Yes	Yes	Yes	Yes	Yes	Yes	Yes	Yes
Year FE	Yes	Yes	Yes	Yes	Yes	Yes	Yes	Yes	Yes
Controls	Yes	Yes	Yes	Yes	Yes	Yes	Yes	Yes	Yes
Observations	837	837	837	837	837	837	837	837	837
F	3.41	0.53	1.02	2.71	3.71	2.79	10.8	2.43	1.04

Note: clustered standard errors are displayed in parenthesis. Control variable is median household income. The average census tract in the average year experienced 11.32 events of private conflict, 7.68 events of public social disorder, and 28.58 events of public violence per 1,000 residents.

Significance levels:

* p<0.05;

** p<0.01;

*** p<0.001.

### Robustness checks

The intent here has been to test whether Airbnb activity in a neighborhood impacts crime, but there is an alternative reverse effect interpretation to our results that need to be considered: That crime leads to Airbnb listings, possibly by deterring property owners from renting long-term or living there themselves–could be true. Rejecting the reverse causality in the DID models is often carried out by testing the pre-treatment parallel trends. However, directly applying the standard tests for parallel trends, such as event-study analysis, is not possible here, because on the one hand, the treatment variable (Airbnb Presence) is both continuous and staggered which makes event-study analysis less reliable and difficult to interpret. On the other hand, our data starts from 2011 where Airbnb had already been present in many neighborhoods (See the Section on *Measuring Airbnb Presence*), preventing us from reliably transforming the treatment into a binary variable that could be used in subsequent event-study analysis (similar to [35]). Because of these reasons and to confirm the direction of causality, we took two additional steps. In the first step, we reran our models with the Airbnb measures from one and two years after the year of the crime measures (See the Methods section.). This method follows the logic of Granger Causality and was popularized by [36] in assessing the impact of unjust dismissal doctrine on outsourcing. Moreover, a recent work by Schmidheiny and Siegloch [37] shows that the event-study analysis and a version of the lag/lead model are equivalent for the case of DID with discrete treatments.

**Fig 4:**
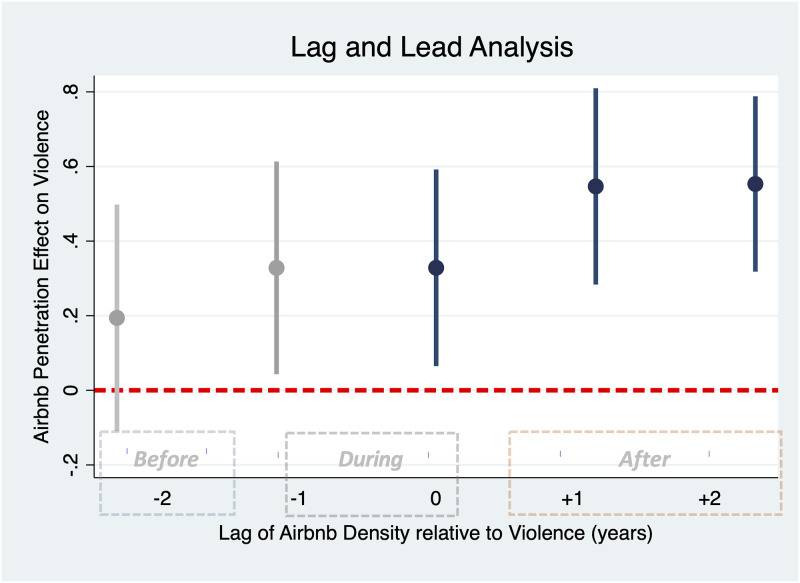
Result of the lag and lead analysis. The figure shows the DID regression coefficients and the corresponding standard errors for the effect of Airbnb density on violence, before, during, and after the effect. Results confirm the direction of causality from Airbnb penetration on violent crimes and show that Airbnb penetration has a significant positive effect on violence, especially with a time delay, but the opposite is not true, as evident from the non-significant effect of a 2-year lead in Airbnb penetration on criminal activities. Complete results are presented in the SI document.

Fig 4 shows a graphical representation of the DID regression coefficients and associated error bars for violent crimes for different time lags(-2 years to +2 years) of Airbnb penetration measure(Full results reported in the SI). The coefficient for two years prior to the treatment (the two-year lead) saw no significant effect on crime, suggesting that with sufficient lead time, these results are consistent with an interpretation of Airbnb’s presence impacting crime and not the reverse.

The one-year lead model still showed an effect of Airbnb penetration on violence, though attenuated relative. This is not entirely surprising since first of all, the treatment variable is continuous, which—unlike [36]—makes it challenging to clearly separate the treatment year from the immediate prior year (the year with one year lead). Moreover, that crime data are aggregated at a yearly basis and our model cannot differentiate between criminal activities at the beginning and end of the year. These reason suggests that due to the resolution and continuous nature of the data, the one year lead is colinear with the zero lead year and can be interpreted, in part, as a period *during* treatment, as marked in the figure. Thus, we need to consider the coefficient for two years prior to the treatment to be able to reject the possibility of reverse causality.

A second and related concern could be the potential bias due to omitted variables. Though the DID models control for the initial conditions of neighborhoods, they do not necessarily control for trends in these variables that parallel the increases in both Airbnb presence and crime. For example, there is some evidence that gentrifying neighborhoods experience increases in certain types of crime [38], and Airbnb listings have also been associated with gentrification [39]. To address this concern and as the second robustness check steps, we reran the models incorporating shifts in four demographic factors–percentage Black residents, percentage Hispanic residents, median income, and homeownership rate–that are often correlated with crime (and are in our data) or believed to be correlated with short-term rentals (e.g., resident-owners are less likely to put their homes up for short-term rental on a regular basis as they live there). We did this by assigning indicators from American Community Survey’s five-year estimates for 2009–2013 to data for 2011–2013, and estimates for 2014–2018 to data for 2014–2017. This is consistent with guidance to not include overlapping estimates in a single analysis [40]. These models did not impact any of the significant effects from the original set of models, indicating our findings were robust to shifts in demographics.

## Discussion and conclusion

This study tested the hypothesis that the arrival and growth of Airbnb, or home-sharing platforms in general, may increase crime and disorder in neighborhoods, focusing specifically on private conflict, public social disorder, and violence. We find that the answer is rather nuanced. Airbnb prevalence in a neighborhood appears to be associated with increases in violence, but not with public social disorder or private conflict. Interestingly, the effect on violence was only consistent visible for the measure of Airbnb penetration–or the extent to which buildings in the neighborhood have one or more listings (and for the measure of density, or the listings per household in the two-year lags). It was never present for overall usage, or the estimated quantity of Airbnb guests. Further, the effect of penetration on violence appears to emerge and strengthen over multiple years.

The specific findings suggest that the impacts of short-term rentals on crime are not a consequence of attracting tourists themselves. Instead, the results point to the possibility that the large-scale conversion of housing units into short-term rentals undermines a neighborhood’s social organization, and in turn its natural ability of a neighborhood to counteract and discourage crime, specifically violent crime. Further, the lagged effects suggest a long-term erosion of the social organization, which would stand in contrast to the more immediate impacts that the presence of tourists would be expected to have. We of course have not directly tested whether social organization is indeed the intervening variable, but it seems clear that the issue is not the tourists themselves but something about how the extreme transience of a short-term rental unit fails to contribute to critical neighborhood social dynamics. We do note that the effects were exclusively on public violence, apart from penetration predicting higher private conflict in the two-year lag. This observation might be for a few reasons. First, social organization is often argued to be particularly important for managing behaviors in public spaces relative to private ones [18]. In addition, public social disorder as measured here, which includes public drunkenness, panhandling, and loitering, is heavily concentrated in Boston’s commercial districts. Thus, such events may be unlikely in residential neighborhoods even with the erosion of social organization. The lack of effects on social disorder, especially drunkenness, might also be taken as additional evidence that tourists staying in short-term rentals are not systematically bringing nuisances to the neighborhood.

The results have important practical implications. To our knowledge, this paper is the first study to robustly test this particular externality of Airbnb at the neighborhood level. Airbnb-related crimes are viewed as a possible consequence of the home-sharing platform because the costs of these incidents are not addressed by the transactions between Airbnb hosts and guests. Instead, these costs are shouldered by increased expenditures for law enforcement and disturbances to neighbors. It is striking to see that the issue is not the visitors themselves but the conversion of units into short-term rentals. In a certain light, this observation is analogous to the effect of Airbnb on housing prices [31, 41–43]. In the one case, Airbnb has removed material capital from the market, raising prices for renters; in the other, Airbnb removes social capital from the neighborhood in the form of stable households, weakening the associated community dynamics.

The apparent unimportance of the tourists themselves might come as something of a surprise given the conceptual and empirical support for the impacts of tourism on crime. It suggests multiple potential explanations. First, although Airbnb has seen notable growth, it might not bring a sufficient quantity of tourists to a neighborhood to have a sustained impact. If there are only a handful of tourists in a neighborhood, the opportunity might not be rich enough to attract predatory crime. Given that we do not expect that other cities have markedly higher Airbnb presence than Boston, we believe this interpretation is extensible to other locales. Second, Airbnb travelers may behave differently in “true” tourist areas than when in the residential neighborhood they are staying in, which in turn could mean that they are less likely to be disorderly or to call attention to themselves as suitable targets.

We note two limitations to our research that call for future studies. First, we have tested this hypothesis in a single city, owing to the availability of both Airbnb listings and 911 dispatches for Boston. Future studies should replicate this analysis in other cities, especially those of different sizes or demographic makeup. Second, we examined a single, hypothesized negative externality of short-term rentals. It does not on its own tell the whole story. Airbnb might have other impacts on neighborhoods–both good and bad. These other relationships require further empirical investigation. Currently, a number of papers have explored how urban planners and policy-makers could respond to potential externalities imposed by Airbnb on urban neighborhoods [44–46], and such efforts will be better informed as we better understand the multifaceted impacts Airbnb can have.

## Supporting information

S1 File (PDF)

S1 Data Airbnb and crime data. (CSV)
