# Correction: Airbnb and neighborhood crime: The incursion of tourists or the erosion of local social dynamics?

**DOI:** 10.1371/journal.pone.0261886

**Published:** 2021-12-21

**Authors:** The PLOS ONE Staff

## Notice of republication

This article was republished on July 21, 2021 to correct errors in the article text that were introduced during the typesetting process. The publisher apologizes for the error. The originally published, uncorrected article and the republished, corrected articles are provided here for reference.

## Supporting information

S1 File Originally published, uncorrected article. (PDF)

S2 File Republished, corrected article. (PDF)
