# Supplementary material for: Airbnb and neighborhood crime: The incursion of tourists or the erosion of local social dynamics?
Source: PLoS One. 2021 Jul 14;16(7):e0253315. doi: 10.1371/journal.pone.0253315 (PMC8279333; doi:10.1371/journal.pone.0253315)
Supplement: S1 File — (PDF) [file pone.0253315.s001.pdf]

Supplementary Information for the paper

Airbnb and Neighborhood Crime: The Incursion of Tourists or the Erosion of Local Social Dynamics?

Laiyang Ke[1,3], Dan O'Brien[1,3], and Babak Heydari[2,1*+]

[1]Northeastern University, School of Public Policy and Urban Affairs, Boston, 02115, USA
[2]Northeastern University, Department of Mechanical and Industrial Engineering, Boston, 02115, USA
[3]Northeastern University, Boston Area Research Initiative (BARI), Boston, 02115, USA

A. Measures of Disorder and Violent Crime

Levels of disorder and crime were calculated from dispatches made by the City of Boston's 911 system. The system generates 700,000 dispatches annually, 91% of which could be referenced to an address or intersection that could be uniquely identified in the list of known locations maintained by the City of Boston (see section on Geographical Coordination of Data). Importantly, these locations are the location where services were required, not necessarily the location from which the request was made. All records contain the date and time the request was received as well as a "case type" drawn from a standardized list to categorize a request at the time of receipt according to the nature of the issue and the services required. Previous work with Boston's 911 archives used confirmatory factor analysis to develop groupings of case types that act as two indices of social disorder and one of violent crime. (O'Brien and Sampson 2015)[1]: public social disorder, such as panhandlers, drunks, and loud disturbances; private conflict arising from personal relationships (e.g., landlord-tenant conflicts); and public violence that did not involve a gun (e.g., fight); and prevalence of guns, as indicated by shootings or other incidents involving guns. Table S1 reports constituent case types for each index and their frequencies for 2011.

[1]The confirmatory factor analyses were based on counts of events of case types for census block groups, maximizing the extent to which case types included in a single category of events (e.g., private conflict) were co-incident at this level of geography.

**Public Violence**		**Public Social Disorder**		**Private Conflict**	
Case Type	Count (2011)	Case Type	Count (2011)	Case Type	Count (2011)
Assault and battery in progress	2181	Intoxication: individual	975	Breaking and entering in progress	1426
Assault and battery report	1565	Drunks causing disturbance	759	Landlord/tenant trouble	667
Armed robbery	350	Panhandler	573	Vandalism report	3502
Emotionally disturbed person: violent or injured	5896	Sex offense/lewd behavior	657	Violation of restraining order	972
Fight	4623	Vandalism in progress	657		
Person with knife	687				

B. Geographic Coordination of Data

The City of Boston's Street and Address Management (SAM) system and Tax Assessor track all properties (i.e., the smallest ownable unit) and land parcels (i.e., geographically-bounded lots that contain one or more properties). SAM also contains a list of street intersections. It is worth noting that this includes all addresses, even if there is no building present (e.g., residential parking lot). Together these form the basis of the Boston Area Research Initiative's Geographical Infrastructure for Boston (GI; O'Brien et al. 2019), which condenses them slightly by combining distinct land parcels with the same postal address that are sufficiently close to each other to be impossible to differentiate. These land parcels are then mapped to U.S. Census TIGER line street segments (i.e., the undivided length of street between two intersections or an intersection and a dead end) and nested within census geographies. This infrastructure if the basis for all analyses in the paper. 911 dispatches typically reference "addresses," which are most consistent with land parcels. They were immediately geocoded to a location in the SAM system by municipal servers until June 2014, which were then directly incorporated into the GI. After that time, a new system was introduced and the coordinates of the 911 calls were projected into latitude and longitude. These were then spatially joined to the nearest land parcel in the GI. Neighborhood measures were than calculated by tabulating the number of social disorder and crime events occurring in each category at each parcel and intersection within a given census tract, divided by the total population in thousands (thereby calculating a rate per 1,000 residents). Airbnb listings come with a "fuzzed" latitude and longitude in the vicinity of the precise address of the listing. InsideAirbnb.com, the organization that scrapes and shares the listings publicly, indicates that the fuzzed coordinates are 0-450 ft. from the actual address. We spatially joined these points to the containing census tract and to the nearest land parcel in the GI and use them to calculate all three measures of Airbnb prevalence in a neighborhood: usage, or the number of reviews of listings in a census tract; density, or the number of listings in a tract divided by the total number of households; and penetration, or the proportion of parcels with at least on listing. We recognize that Airbnb's fuzzing process introduces error into each of these measures. For usage and density, these errors are probably rather low as most census tracts cover a space with approximate radius of 0.5 miles (2,500 feet), meaning the vast majority of listings will fall in

the correct census tract. For penetration, the assumption that parcels are accurate is a bit more vulnerable. That said, our primary goal here is to capture whether the listings in a given census tract are geographically concentrated in one or two areas versus distributed throughout. As such, this is the best proxy available of penetration throughout the neighborhood as differentiated from density, which can be geographically concentrated as traditionally calculated.

C. Lag and Lead Analysis to Test the Direction of Causality

To test the direction of causality for the results, we use a lag/lead analysis in the spirit of Granger [1, 2]. This is method is is used when the sample includes multiple years and uses both lead and lagged versions of the treatment variable (τ can be both positive and negative).

$$\mathrm{Y}_{i,t} = \alpha + \gamma Airbnb_{i,t-\tau} + \delta X_{i,t} + \eta_i + \beta_t + \varepsilon_{i,t}$$

If the direction of causality is from Airbnb presence to crime, we expect to see significant effect when τ is negative and no significant effect when τ is positive. Furthermore, we expect the magnitude of the effect to increase as τ gets smaller. Below, we present a 5-year lag and lead analysis for all three measures of Airbnb presence, and all three measures of criminal activities.

**Penetration (t+τ)**	**Violence(t)**	**Social Disturbance(t)**	**Private Conflict(t)**
**Penetration(t+2)**	0.1941	0.1132	-0.0345
SE	(0.1535)	(0.1004)	(0.045)
**Penetration(t+1)**	0.32817*	0.059	-0.0184
SE	(0.1439)	(0.0718)	(0.0383)
**Penetration(t)**	0.3284*	-0.00411	0.0048
SE	(0.1331)	(0.07319)	(0.0353)
**Penetration(t-1)**	0.5465***	-0.1145	0.0407
SE	(0.1329)	(0.118)	(0.0394)
**Penetration(t-2)**	0.5532***	-0.1616	0.0968*
SE	(0.1439)	(0.1068)	(0.0406)

Note: clustered standard errors are displayed in parenthesis. Control variable is median household income.
Significance levels: * p<0.05; ** p<0.01; *** p<0.001.

Table 1: 5-year Lag and Lead Analysis for the effect of Airbnb *penetration* on neighborhood violence, social disturbance and private conflict. Results support the direction of causality from Airbnb penetration to an increase in violence. Number of observations are 1171, 1004 and 837, for τ = 0,$\pm$ 1 and $\pm$ 2 respectively.

**Density($t+\tau$)**	**Violence(t)**	**Social Disturbance(t)**	**Private Conflict(t)**
**Density(t+2)**	0.546	0.421	-0.1071
SE	(0.591)	(0.306)	(0.1888)
**Density(t+1)**	0.8975	0.7584	-0.3014
SE	(0.5999)	(0.4732)	(0.1885)
**Density(t)**	1.2257*	0.0799	-0.207
SE	(0.6198)	(0.2842)	(0.2065)
**Density(T-1)**	1.40066*	-0.4269	-0.1112
SE	(0.6122)	(0.2922)	(0.2266)
**Density(t-2)**	1.1668*	-0.8863	0.3811
SE	(0.5279)	(0.471)	(0.2145)

Note: clustered standard errors are displayed in parenthesis. Control variable is median household income.
Significance levels: * p<0.05; ** p<0.01; *** p<0.001.

Table 2: 5-year Lag and Lead Analysis for the effect of Airbnb *density* on neighborhood violence, social disturbance and private conflict.

**Usage($t+\tau$)**	**Violence(t)**	**Social Disturbance(t)**	**Private Conflict(t)**
**Usage(t+2)**	1.67	2.98	-0.25
SE	(2.25)	(2.41)	(-0.96)
**Usage(t+1)**	1.19	1.068	-1.11
SE	(2.2)	(1.39)	(0.9)
**Usage(t)**	2.54	-0.44	0.01
SE	(2.1)	(1.12)	(0.76)
**Usage(t-1)**	3.67	-1.125	6.28E-02
SE	(2.17)	(1.62)	(0.901)
**Usage(t-2)**	3.67	-3.56	1.36
SE	(2.72)	(2.85)	(1.26)

Note: clustered standard errors are displayed in parenthesis. Control variable is median household income.
Significance levels: * p<0.05; ** p<0.01; *** p<0.001.

Table 3: 5-year Lag and Lead Analysis for the effect of Airbnb *usage* on neighborhood criminal activities.

References

[1] Joshua D Angrist and Jörn-Steffen Pischke. *Mostly harmless econometrics: An empiricist's companion.* Princeton university press, 2008.

[2] Clive WJ Granger. "Investigating causal relations by econometric models and cross-spectral methods". In: *Econometrica: journal of the Econometric Society* (1969), pp. 424–438.
